# The response and survival of children with recurrent diffuse intrinsic pontine glioma based on phase II study of antineoplastons A10 and AS2-1 in patients with brainstem glioma

**DOI:** 10.1007/s00381-014-2401-z

**Published:** 2014-04-10

**Authors:** Stanislaw R. Burzynski, Tomasz J. Janicki, Gregory S. Burzynski, Ania Marszalek

**Affiliations:** Burzynski Clinic, 9432 Katy Freeway, Houston, TX 77055 USA

**Keywords:** Antineoplastons A10 and AS2-1, Brainstem glioma, Diffuse intrinsic pontine glioma, Recurrent glioma, Phase II clinical trial

## Abstract

**Background:**

Brainstem gliomas (BSG) are relatively rare tumors of which recurrent pediatric diffuse intrinsic pontine gliomas (RPDIPG) comprise a distinct group. Numerous trials have been conducted on RPDIPG, none of which have resulted in identifying any proven pharmacological treatment benefit. This study included 40 patients diagnosed with different types of BSG, but it was decided to describe first the encouraging results in the most challenging group of RPDIPG.

**Materials and methods:**

This single-arm phase II study evaluated the efficacy and safety of the combination of antineoplastons A10 and AS2-1 (ANP) in patients with RPDIPG. Seventeen patients (median age 8.8 years) were enrolled, and all were diagnosed with RPDIPG. ANP was administered intravenously daily. Efficacy analyses were conducted in this group of patients.

**Results:**

In this group, complete responses were observed in 6 % of patients, partial responses in 23.5 %, and stable disease in 11.8 %. Six-month progression-free survival was 35.3 %. One-year overall survival was 29.4 %, 2 years 11.8 %, and 5, 10, and 15 years 5.9 %. One patient with DIPG is alive over 15 years post-treatment. Grade 3 and higher toxicities including hypokalemia and fatigue occurred in 6 %, hypernatremia in 18 %, fatigue and urinary incontinence in 6 %, and somnolence in 12 %. In a single patient, grade 4 hypernatremia occurred when he was on mechanical ventilation. He was disconnected from the ventilator and died from brain tumor according to the attending physician. Responding patients experienced improved quality of life.

**Conclusion:**

The results suggest that ANP shows efficacy and acceptable tolerability profile in patients with RPDIPG.

## Introduction

Brain tumors in children account for 20 % of all neoplasms [1]. Brainstem gliomas are relatively rare tumors which constitute 1.6 % of primary brain and CNS tumors by site [2]. In children, high-grade gliomas—anaplastic astrocytomas (AA), glioblastoma multiforme (GBM), and diffuse intrinsic pontine gliomas (DIPG)—are the most aggressive neoplasms and the leading causes of cancer-related mortality [1, 3, 4]. Based on the most recent studies, pediatric DIPG forms a distinct group which is characterized by recently identified mutations in the histone H3.3 (*H3F3A* gene) [4]. Numerous clinical trials have been carried out in patients with DIPG, but no pharmacological agents have to date proven efficacious—leaving standard radiation therapy (RT) as the mainstay of treatment [1, 3]. The overall outlook is poor and nearly all children eventually die. In most studies, the median survival time is shorter than 1 year and less than 10 % of patients survive over 2 years [1].

**Fig. 1:**
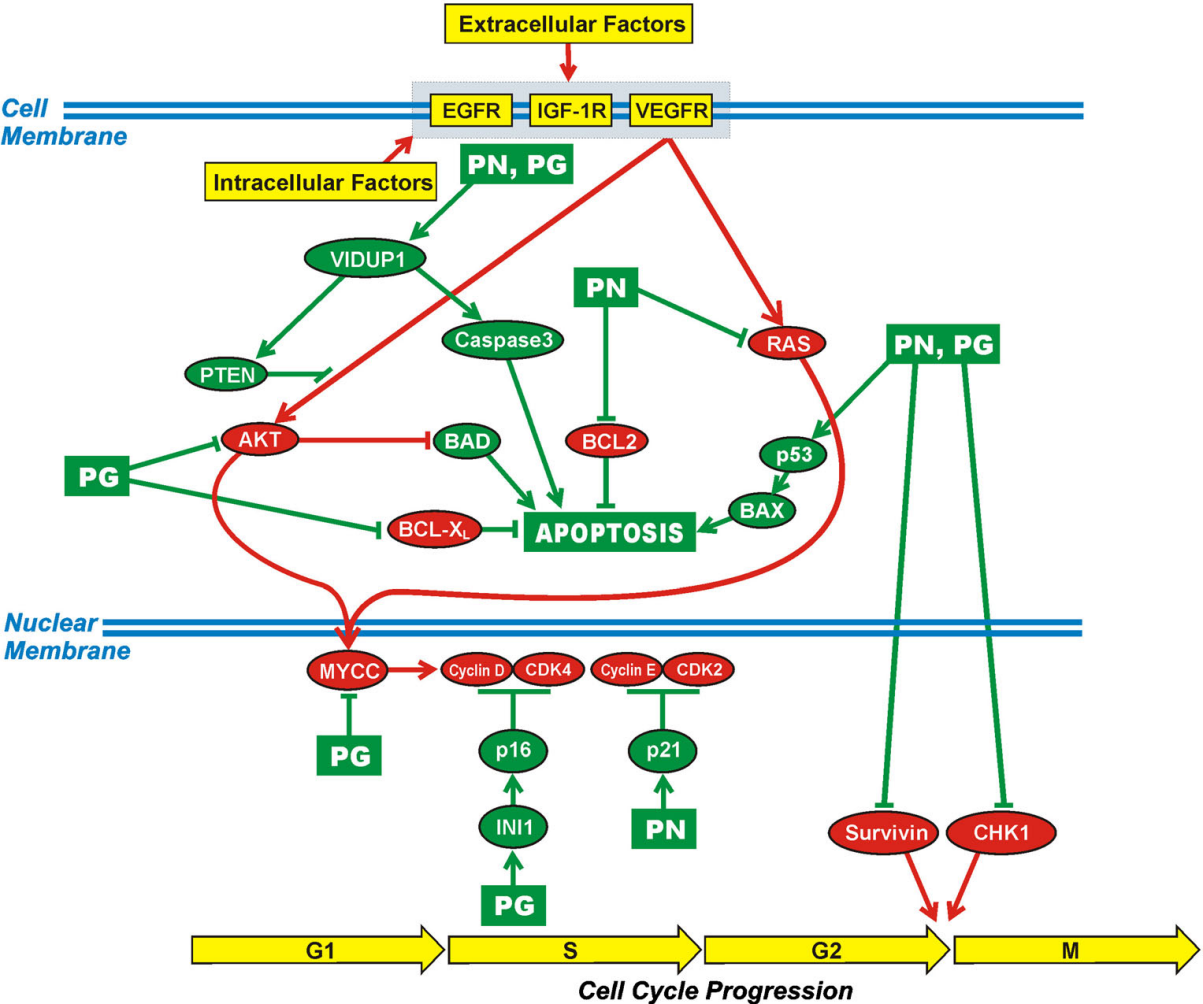
Proposed mechanism of action of antineoplastons A10 and AS2-1. The ingredients of antineoplastons A10 and AS2-1, PN and PG, affect signal transmission through AKT and RAS pathways, promote apoptosis, and interrupt cell cycle progression at G1/S and G2/M checkpoints

Antineoplastons A10 and AS2-1 (ANP) are synthetic amino acid derivatives. A10 is a formulation consisting of a 4:1 ratio of phenylacetylglutaminate sodium (PG) and phenylacetylisoglutaminate sodium (isoPG). AS2-1 is a formulation with a 4:1 ratio of phenylacetate sodium (PN) and PG [5]. The proposed antineoplastic activity of ANP in gliomas is shown in Fig. 1 and consists of specific effects on the *AKT2* and *RAS* pathways and tumor suppressor genes *TP53* and *p21* and on apoptosis [6–16].

Genome-wide analyses have been conducted to identify recurrent amplifications of receptor tyrosine kinases (RTKs) and cell cycle regulatory genes in DIPGs and indicated substantial tumor heterogeneity and involvement of *RAS* and *AKT* signaling pathways [17, 18]. Successful treatment of a heterogeneous tumor may require inhibitors that affect not only multiple targets, but also multiple pathways, such as ANP.

Phase I studies of injections of antineoplastons A10 and AS2-1 (ANP) as well as prior phase II studies included pediatric patients [5, 19–22]. In two of these studies, complete responses (CR) were observed in pediatric DIPG [5, 23]. In our phase II study (BT-03) of ANP of 20 subjects with astrocytomas, conducted in 1988, CR were documented in two cases of DIPG in young adults [20, 21]. One of these patients is currently surviving tumor free for over 25 years after being treated with ANP for DIPG, which recurred after radiation therapy. These positive preliminary data prompted us to design and conduct a single-arm phase II study of ANP to assess the efficacy and safety of this treatment in pediatric patients diagnosed with recurrent DIPG (RPDIPG).

## Patients and methods

### Patient population

The protocol permitted admission of patients with brainstem glioma for whom standard curative treatments are not available. A total of 40 patients were enrolled, but the scope of this paper has been limited to the 17 RPDIPG patients.

Recruited patients were all over 4 years of age and had radiologic evidence of brainstem glioma by gadolinium-enhanced MRI performed 14 days prior to being enrolled in the study. Based on MRI only, DIPG can be diagnosed if the tumor has an epicenter in the pons and involves more than 50 % of the pons. Patients with neurofibromatosis are not covered by this definition and were not included. The tumors involving less than 50 % of the pons or exophytic were classified as DIPG if they had anaplastic or GBM histology [24–26].

Eligibility criteria included a Karnofsky Performance Status (KPS) of 60–100. The subjects were required to have relatively normal hematopoietic and hepatic function with white blood cell count (WBC) over 1,500/mm^3^ and platelet count over 50,000 mm^3^, no evidence of hepatic or renal insufficiency, and a total bilirubin and serum creatinine of no higher than 2.5 mg/dL. SGOT and SGPT were to be no higher than 5× the upper limits. At least 8 weeks must have elapsed since the last dose of RT, and at least 4 weeks since the last dose of chemotherapy (6 weeks for nitrosoureas), or immunotherapy. The use of corticosteroids was permitted to reduce symptoms and signs attributed to cerebral edema, but it was recommended that the smallest doses compatible with the preservation of optimal neurologic function be used. Confirmation of the pathologic diagnosis by an outside pathologist was also required.

The exclusion criteria included serious active infection, fever, or other serious concomitant disease that would interfere with the evaluation of the treatment (e.g., severe heart or lung disease). There were no exclusion criteria based on tumor size, multifocality, or leptomeningeal involvement.

All study subjects and/or guardians read, understood, and signed written informed consent prior to enrollment. This study was conducted in accordance with the US Code of Federal Regulations, Title 21, Parts 11, 50, 56, and 312; the Declaration of Helsinki (1964) including all amendments and revisions; the Good Clinical Practices: Consolidated Guideline (E6); International Conference on Harmonization; and the FDA’s Guidance for Industry. The study was sponsored by the Burzynski Research Institute (BRI) and conducted by the Burzynski Clinic (BC) in Houston, TX. The patients did not pay for the investigational agents.

### Study design

The study was designed as a single-arm, two-stage, phase II trial of ANP as monotherapy. The study was listed by the National Cancer Institute (NCT00003459). It was supervised by an independent Institutional Review Board (BRI-IRB, BC-BT-11).

The study was performed according to Protocol BT-11 which was submitted to the FDA under the IND 43,742. Subsequently, the protocol was amended by BRI several times; however, none of the amendments altered the aim or design of the original study objectives/outcomes.

### Statistical considerations

#### Sample size

The sample size was calculated based upon the previously used method described by Chang et al. [27]. A response rate to ANP of ≥10 % was considered “of interest,” and the primary endpoint was to determine the overall response rate which was confirmed CR or partial response (PR) to ANP therapy. Objective response (OR) and progression-free survival (PFS) were measured from the first day of ANP administration. The distributions of survival and treatment failure were estimated by Kaplan-Meier analysis. As mentioned before, this paper described 17 RPDIPG patients out of the total of 40 brainstem glioma patients.

#### Treatment

The median maximum dose of ANP A10 was 8.14 g/kg/day (5.69–15.87) and for AS2-1 0.42 g/kg/day (0.21–0.58). The duration of IV ANP therapy ranged from 5 to 178 weeks with a median of 61 weeks.

ANP A10 and AS2-1 were delivered via a dual-channel infusion pump and single-lumen subclavian catheter (Broviac or Groshong) every 4 h. On the first day of administration of ANP, the flow rate of the pump was maintained at 25 mL/h. Beginning from the second day, individual injections were given at 100 to 250 mL/h depending on the patient’s age and tolerance.

Approximate guidelines for flow rates are provided.

1. Four to 7 years old—flow rate 100 mL/h
2. Seven to 10 years old—flow rate 150 mL/h
3. Ten to 16 years old—flow rate 200 mL/h
4. Sixteen to 18 years old—flow rate 250 mL/h

On the first day of treatment, the pump was loaded with 60 mL of A10 (0.3 g/mL) and 60 mL of AS2-1 (0.08 g/mL). The volume of each injection was 10 mL administered every 4 h, six times a day. Beginning from the second day of treatment in children younger than 12 years of age, the dose of each injection was increased on a daily basis in increments of 10 mL until the highest tolerable dose or effective dose was reached. For children 12 years of age or older, the dose of A10 was escalated in increments of 20 mL daily, and over 16 years of age, in increments of 40 mL. When the study subject reached the highest tolerated dose, the “escalation phase” of the treatment stopped.

In summary, there were three different volumes of daily dosage increments of ANP depending on the patient’s age: 10 mL daily dose increase per injection for children younger than 12 years of age, 20 mL for children between 12 and 15 years of age, and 40 mL for patients over 16 years of age. The complicated part of the regimen, which is escalation of dosage, was finished during the patient’s treatment at BC under careful supervision of the sub-investigator assigned to the case. Upon returning home, the patient is given a regimen that consisted of administration of the fixed dose (volume) of ANP through an automatic pump six times a day. The pump was typically loaded with ANP once a day and the rest of the administration was done automatically. It was possible to disconnect the pump in between IV boluses given every 4 h to permit patient’s physical activities. The single dosing could be completed within 1 h which would leave 3 h until the next dose, and during this time, the pump could be disconnected. Long-term observation of the patients confirmed that the regimen created a limited burden on the quality of life. After symptomatic improvement, the children were able to attend school and graduate from it. The subject continued the daily administration of six doses of A10 and AS2-1 (every 4 h via an automated pump) until a response to the treatment was determined.

The rationale for using two formulations of ANP was based on prior clinical trials, pharmacokinetic studies, and laboratory research [5]. The escalation of the dosage of ANP was recommended based on the positive results of previous studies carried out to determine whether patients were able to tolerate large volume infusions of intravenous fluids associated with higher doses of ANP [5]. As a safety precaution, it was recommended that the escalation of the dosages would continue through phase II and phase III trial programs.

Medications that were considered necessary for the patients’ welfare and that did not interfere with the evaluation of treatment were given at the discretion of the investigator. The use of corticosteroids was carefully monitored. Treatment with other antineoplastic or immunomodulatory agents was not permitted during the study. Subjects received full supportive care, including transfusions of blood products and antibiotics when appropriate.

The initial 3 weeks of therapy was administered by BC staff on an outpatient basis, in Houston, TX. The treatment did not require hospitalization. Subjects and/or their legal guardians were trained by clinic staff to self-administer ANP therapy during this time. Starting on week 4, ANP therapy was administered at home with 24-h support available via phone. Treatment and monitoring of the subject’s condition, once released to self-administered therapy, continued under the supervision of the subject’s local attending physician (physician who signed the FDA form 1572).

#### Evaluation and follow-up

Prior to the start of treatment, a gadolinium-enhanced MRI measured all contrast-enhancing lesions. The products of the two greatest perpendicular diameters of all lesions were calculated and totaled, providing a baseline evaluation for each study subject. The tumor measurements were based on contrast-enhanced lesions, but the nonenhancing lesion and overall tumor size were also measured including T2 and FLAIR images [26, 28]. CR required the disappearance of all enhancing lesions sustained for at least 4 weeks, with only physiologic replacement doses of steroids acceptable. Positron emission tomography (PET) shows resolution of hypermetabolic lesions. PR required 25 % or higher decrease of the sum of the products of the two largest perpendicular diameters of enhancing lesions and stable or reduced corticosteroid doses. PD was determined when there was over 25 % increase of enhancing lesions or new lesions, and stable disease (SD) was the status between PR and PD. In the case of SD, the duration was measured from the time therapy commenced. The results of all MRI and PET scans were verified by radiologists not affiliated with BRI or BC, and their determination of response was accepted. Complete and partial responses were verified by central radiology review.

Blood and urine tests (complete blood count with differential, platelet count, reticulocyte count, and serum chemistry), anticonvulsant serum levels, prothrombin time, and partial thromboplastin time were carried out on all subjects prior to the start of treatment to establish baseline values. The additional pretreatment measurements included KPS, vital signs, clinical disease status, demographics, medical history and current medications, physical examination with neurologic emphasis, chest X-rays, and EKG. Toxicity was evaluated in all patients enrolled in the study. Data on adverse drug experiences (ADEs) were collected during the initial 3 weeks of ANP therapy by clinic staff at the BC. MRIs were repeated at least every 8 weeks during the first 2 years unless the patient’s condition or confirmation of response required an MRI within 4 weeks. PET scans were performed as necessary. When study subjects transitioned to home-based therapy administration under the care of a local physician, clinic staff made daily telephone contact for the first 2 months to ensure protocol compliance, to resolve any issues with therapy administration, and to continue assessing ADEs. Weekly contact was made starting in the third month. Continued patient treatment with ANP was determined on at least a weekly basis and based upon the trial protocol, patient health status, and the response to treatment.

The records of daily administration of ANP were maintained and carefully checked for drug accountability. The ADEs were graded according to the Common Terminology Criteria for Adverse Events (CTCAE v.3). Pharmacokinetic studies have been previously carried out and were not included in this study. Based on a prior study, there was no indication of interference with essential supportive medications, in particular, antiseizure drugs.

## Results

### Patient demographics

The enrollment of subjects diagnosed with RPDIPG commenced on March 13, 1996 and continued through December 14, 2006. As of September 12, 2007, all subjects were removed from the therapy, as a consequence of either a CR, subject request, PD, or worsening clinical condition.

Demographics for the subjects are summarized in Table 1. Patient demographics did not change during the study and were comparable to the other studies conducted on brainstem glioma. The tumor measurements on MRI T2-weighted images determined from 61 to 100 % involvement of the pons (median 85 %). There were one GBM and one AA as well as two cases of pilocytic astrocytoma, grade 1 in this group. The data on confirmation of diagnosis and tumor recurrence, confirmation of response, and change in tumor measurements during treatment are provided in Table 2.

**Table 1:** Study population demographics

Characteristics	Recurrent pediatric DIPG, *n* = 17
Demographics	
Age (years): median	8.8
Range	4.5–18.5
Gender: male	8
Female	9
Ethnicity	C—15, H—1, O—1
Tumor histopathology	
Glioblastoma multiforme	1
Anaplastic astrocytoma	1
Astrocytoma, grade I	2
Karnofsky Performance Score	
Median (%)	BT / AT
	70 / 80
60	6 / 4
70	5 / 2
80	5 / 6
90	0 / 1
100	1 / 2
Duration of symptoms prior to enrollment	
Less than 6 months	18 %
Greater than 6 months	82 %
Prior treatment	
SU	6 %
RT	29 %
CH	6 %
CH + RT	53 %
SU + CH + RT	6 %
One-regimen CH	82 %
Two-regimen CH	0
Three-regimen CH	18 %

*AT* at the end of treatment, *BT* at treatment start, *C* Caucasian, *CH* chemotherapy, *DIPG* diffuse intrinsic pontine glioma, *H* Hispanic, *O* Oriental, *RT* radiation therapy, *SU* surgery

**Table 2:** Confirmation of diagnosis, recurrence, and response

Confirmation of diagnosis					Treatment	Confirmation of recurrence			Confirmation of response to ANP		Tumor measurements		
Pathology			Radiology										
Patient	Place and date	Diagnosis	Place and date	Diagnosis		Place and date	Assessment	Pons T2 %^a^	Place and date	Assessment	MRI enhancing	MRI nonenhancing	PET
1	Cancer Institute/July 12, 1994	PA	Cancer Institute/July 1994	DIPG	S, RT, CH	External Radiology, CRR/March 25, 1996	Recurrence	61	External Radiology, CRR	PR	Decreased >50 %	Decreased	
2			University Hospital/August 17, 1995	DIPG	RT, CH	External Radiology/March 13, 1996	Recurrence	75	None	NE	UNK	UNK	
3			External Radiology/October 1, 1995	DIPG	RT, CH	External Radiology/June 17, 1996	Recurrence	65	External Radiology/November 20, 1996	SD	Stable	Decreased	
4			University Hospital/March 8, 1996	DIPG	RT, CH	External Radiology, CRR/August 29, 1996	Recurrence	100	External radiology, CRR/December 5, 1996	PR	Decreased >50 %	Decreased	
5			University Hospital/March 28, 1996	DIPG	RT	External Radiology/October 10, 1996	Recurrence	100	External Radiology/January 13, 1997	PD	Increased >40 %	Stable	
6			External Radiology/January 31, 1996	DIPG	RT	External Radiology/October 25, 1996	Recurrence	100	External Radiology/January 13, 1997	PD	Increased >50 %	Stable	
7			External Radiology/August 26, 1997	DIPG	CH	External Radiology/October 6, 1997	Recurrence	88	External Radiology/January 21, 1998	SD	Stable	Stable	
8	University/February 1, 1993	PA	University Hospital/January 28, 1993	DIPG	2S	External Radiology, CRR/March 1, 1998	Recurrence	72	External Radiology, CRR/January 16, 1999 and May 6, 2003	PR, CR	Decreased >50 %	Decreased	Neg
9			University Hospital/November 11, 1997	DIPG	RT, CH	University Hospital/April 2, 1998	Recurrence	100	External Radiology, CRR/October 6, 1998	PR	Decreased >50 %	Decreased	
10			External Hospital/October 2, 1998	DIPG	RT	External Hospital/April 9, 1999	Recurrence	87	External Hospital/July 15, 1999	PD	Increased >25 %	Stable	
11			External Hospital, Neuro-Oncology Tumor Board/September 25, 2001	DIPG	RT	External Radiology/January 8, 2002	Recurrence	92	External Radiology, CRR/May 30, 2002	PR	Decreased >50 %	Decreased	
12	University Hospital/September 4, 2002	AA	University Hospital/March 3, 2002	DIPG	RT, CH	University Hospital/August 29, 2002	Recurrence	85	None	NE	UNK	UNK	
13			University Hospital/December 30, 2002	DIPG	RT, CH	External Radiology/August 19, 2003	Recurrence	81	External Radiology/December 29, 2003	PD	Increased >50 %	Increased	
14			University Hospital/January 6, 2004	DIPG	RT	External Radiology/October 5, 2004	Recurrence	87	External Radiology/January 5, 2005	PD	Increased >25 %	Stable	
15			University Hospital/June 1, 2005	DIPG	RT, CH	External Radiology/September 20, 2005	Recurrence	67	External Radiology/January 31, 2006	PD	Increased >50 %	Increased	
16			University Hospital/March 20, 2006	DIPG	RT, CH	External Radiology/August 9, 2006	Recurrence	71	External Radiology/October 31, 2006	PD	Increased >50 %	Increased	
17	Cancer Institute/August 2, 2006	GBM	Cancer Institute/August 10, 2006	DIPG with leptomeningeal dissemination	RT, 3CH, HDCH	Cancer Institute/October 30, 2006	Recurrence	73	External Radiology/April 2, 2007	SD	Stable	Decreased	

*AA* anaplastic astrocytoma, *ANP* antineoplaston, *CH* chemotherapy, *CR* complete response, *CRR* central radiology review, *DIPG* diffuse intrinsic pontine glioma, *GBM* glioblastoma multiforme, *HDCH* high-dose chemotherapy, *NE* nonevaluable, *Neg* negative, *PA* pilocytic astrocytoma, *PD* progressive disease, *PET* positron emission tomography, *PR* partial response, *RT* radiation therapy, *S* surgical resection, *SD* stable disease, *T2* weighted imaging, *UNK* unknown

^a^Measurements reflect percentages of the product of the two longest perpendicular diameters of the pontine tumor versus the product of the two longest perpendicular diameter of pons of the T2-weighted images

### Efficacy

**Fig. 2:**
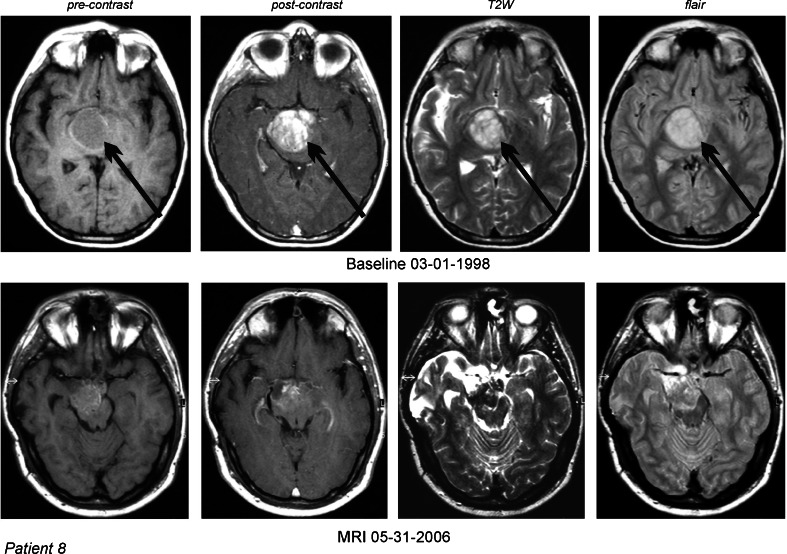
DIPG in a 10-year-old male (case 8) which recurred two times after partial surgical resection. MRI of the head: 1—T1 nonenhanced, 2—contrast-enhanced, 3—T2W, and 4—FLAIR images. PR was documented by the MRI and CR was established by the normalization of the follow-up PET scans. *Arrows* indicate tumors

**Fig. 3:**
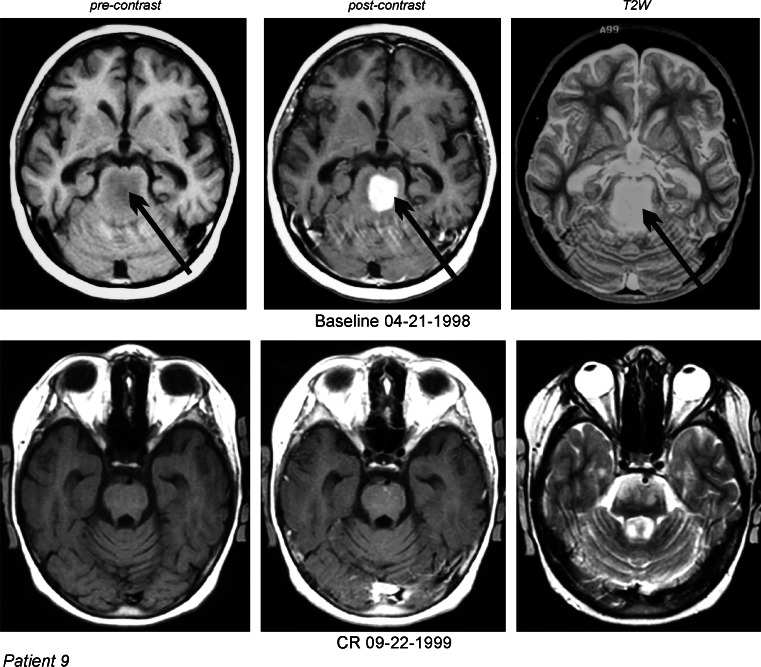
DIPG in a 7-year-old female (case 9) which recurred after radiation therapy and chemotherapy with etoposide. MRI of the head: 1—T1 nonenhanced, 2—contrast-enhanced, and 3—T2W images. MRI documented PR. *Arrows* indicate tumors

In the group of 17 RPDIPG patients, there were five cases of OR (29.5 %): one CR (6 %) and 4 PR (23.5 %). An additional group of two patients (11.8 %) had stabilization of their disease, eight patients (47 %) developed progressive disease, and two cases (12 %) were nonevaluable (NE). Figures 2 and 3 illustrate the response to treatment with ANP in two patients.

### Survival data

**Fig. 4:**
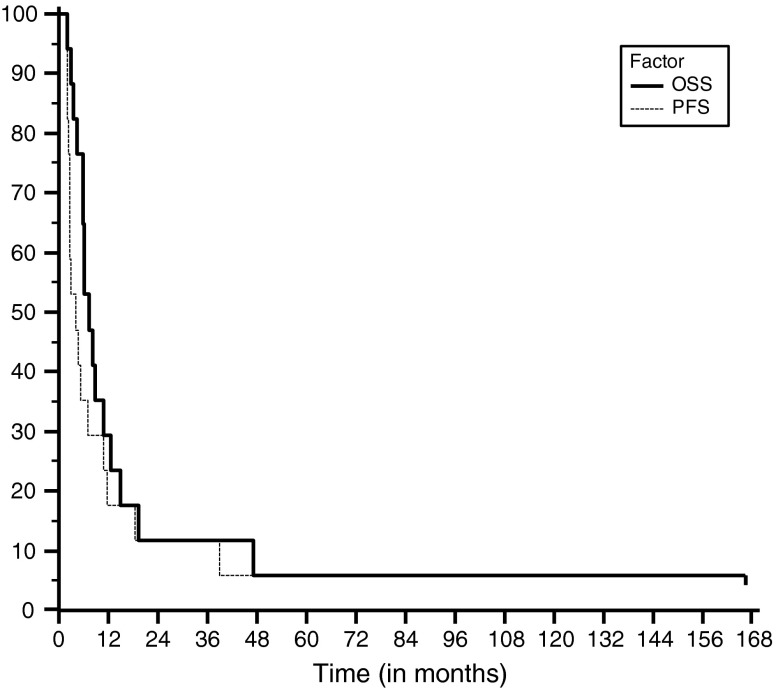
The Kaplan-Meier survival curves from the start of treatment for recurrent pediatric DIPG

The Kaplan-Meier OS and PFS curves are shown in Fig. 4. Estimated overall survival at 10 and 15 years is 5.9 % up to July 2013. The survival is 29.4 % at 1 year and 11.8 % at 2 years. One patient remains alive over 15 years from the start of treatment.

### Safety and adverse events

#### Study population

Safety assessments were analyzed based upon the total number of enrolled patients (*n* = 17). Grade 4 toxicities including hypokalemia and fatigue occurred in 6 % and hypernatremia in 18 % of the group. Grade 3 fatigue and urinary incontinence occurred in 6 % and somnolence in 12 %. In patient 2 (Table 2), grade 4 hypernatremia was detected when he was on mechanical ventilation in the intensive care unit. This patient received terminal care and was disconnected from the ventilator upon request of the members of his family. His attending physician determined brain tumor as the cause of death. No chronic events occurred. Responding patients experienced improved quality of life. Brain tumor patients frequently receive corticosteroids as part of their therapeutic regimen to reduce cerebral edema around tumors. The use of corticosteroids, the infusion of large volumes of sodium-containing solutions during ANP therapy, and the brain tumor itself predispose a patient to an increased incidence of serum sodium and potassium concentration abnormalities. As a result, hypernatremia and hypokalemia were reported in a substantial number of patients.

## Discussion

Between 1984 and 2005, 29 studies in DIPG have been reported and were reviewed by Hargrave et al. [1]. The additional clinical trials were reviewed by us previously [3, 29]. These trials did not demonstrate any improvement in efficacy beyond standard radiation therapy which remains the standard treatment. Less than 10 % of children with a diagnosis of DIPG do not survive more than 2 years from diagnosis, and very few live beyond 5 years. In recent years, only seven trials were conducted for a mixed population of pediatric patients, usually with high-grade glioma, with a small percentage of patients diagnosed with DIPG [30–36]. The results from this trial in comparison to other comparable studies are presented in Table 3. Five trials were conducted for recurrent brainstem glioma, usually at the first relapse. The remaining two trials included children with both recurrent as well as newly diagnosed brainstem glioma. Lashford et al. reported data on treatment with RT and temozolomide (TMZ) in 18 eligible patients diagnosed with progressive diffuse intrinsic brainstem glioma with the tumor centered in the pons in 15 subjects [30]. They reported a PR of 6 %, SD of 17 %, and a median survival of 6.2 months. Dreyer et al. evaluated ten brainstem glioma patients with recurrent or progressive disease after conventional RT and chemotherapy [31]. These patients were treated with an analog of daunomycin, idarubicin. No OR was reported, but there were one case of SD and seven cases of PD among eight evaluable patients. Warren et al. used a combination of lobradimil and carboplatin for brainstem glioma at first relapse (12 patients) [32]. There was no OR and the median time to progression was only 84 days. Tipifarnib was used by Fouladi et al. in a phase II trial for recurrent or progressive high-grade glioma including brainstem glioma and PNET [33]. In 41 patients, there were one PR and four cases of SD (11 %). Gururangan et al. used a combination of bevacizumab and irinotecan in children with recurrent DIPG [34]. In the population of 17 patients, there was no OR, but five cases of SD and 13 PD. Median PFS was 2.3 months and 6-month PFS was 9.7 %. Minturn et al. used metronomic oral topotecan in nine children with recurrent brainstem glioma. They reported SD in two patients, but no OR [35]. Warren et al. conducted a phase II study with 06-benzylguanine and TMZ in 16 children with recurrent or progressive brainstem gliomas [36]. No OR was reported and 6-month PFS was zero. These trials failed to prove efficacy for any of the treatment regimens under evaluation and the treatments themselves were associated with substantial toxicity.

**Table 3:** Phase II clinical studies in recurrent or progressive brainstem glioma in children

Author/treatment	Total no. of patients	Prior treatment		Efficacy			PFS		Survival					
		Radiation therapy (%)	Chemotherapy (%)	CR (%)	PR (%)	SD (%)	Median (months)	At 6 months (%)	Median (months)	1 years (%)	2 years (%)	5 years (%)	10 years (%)	15 years (%)
Lashford et al. [30]/temozolomide	18	All	28	0	6	17								
Dreyer et al. [31]/idarubicin	10	96	All	0	0	10								
Warren et al. [32]/lobradimil and carboplatin	12	95	Majority	0	0	0								
Fouladi et al. [33]/tipifamib	41		Recurrent refractory brainstem gliomas^a^	0	2	11								
Gururangan et al. [34]/bevacizumab and irinotecan	17	100		0	0	29		9.7						
Minturn et al. [35]/topotecan	9		Recurrent refractory brainstem gliomas^a^	0	0	22								
Warren et al. [36]/06-benzylguanine and temozolomide	16	100	31	0	0	6		0						
Burzynski et al. [23]/antineoplastons A10 and AS2-1	17	88	65	6	23.5	11.8	4.08	35.3	7.5	29.4	11.8	5.9	5.9	5.9

*CR* complete response, *PR* partial response, *SD* stable disease, *PFS* progression-free survival, *OS* overall survival

^a^No information available on prior treatment

The review of the literature indicates that there are substantial differences in the definitions of DIPG and the evaluations of the responses. In this study, we accepted the criteria used for all COG and PBTC studies. DIPG are predominantly nongadolinium enhancing, hyperintense on T2-weighted images and hypodense on T1. Despite this fact, all studies conducted within the last 10 years used the Macdonald evaluation criteria, based on measurements of contrast-enhancing lesions. It is generally assumed that it would be preferable to have an objective measure of nonenhancing, recurrent tumor, but the opinion of the response assessment in the neuro-oncology (RANO) working group is that this is not possible at the present time due to limitations of technology [26]. Unfortunately, the evaluation of the response based on the MRI could be misleading, and for this reason, we included the measurement of the nonenhancing tumor, changes in the patient’s clinical condition, as well as PFS and OS. It is generally accepted, although not ideal, that the use of overall survival is the most reliable variable [1].

The results of this study compare favorably to the other trials in patients with RPDIPG. The survival data in most of these trials are nonexistent simply because patients do not usually survive more than 6 months. Contrary to the other studies, our PFS at 6 months is 35.3 % and overall survival from the treatment start at 1 year is 29.4 % and approximately 11.8 % at 2 years. Even at 15 years, the overall survival is approximately 5.9 % (patient was diagnosed with pilocytic astrocytoma and had tumor involving 72 % of pons at the baseline). The quality of survival is excellent and median KPS at the conclusion of treatment increased to 80. There are no chronic adverse events, and the moderate percentages of serious acute toxicities reported are manageable.

## Conclusions

The treatment of brainstem gliomas poses the greatest challenge for pediatric neuro-oncologists. This small phase II study of ANP A10 and AS2-1 reports encouraging CR and PR in recurrent pediatric DIPG with promising survival data. Additional phase II studies of ANP in pediatric high-grade glioma and brainstem glioma as well as astrocytoma and optic pathway glioma have been completed and are being prepared for publication.
